# Prosthetic Aortic Valve Endocarditis Due to Candida glabrata Successfully Treated With Intravenous Micafungin Followed by Oral Fluconazole Without Surgery: A Case Report and Literature Review

**DOI:** 10.7759/cureus.79550

**Published:** 2025-02-24

**Authors:** Muhammad Waqar Elahi, Eric Huang, Vikash Kumar, Mona Ghias, David Shi

**Affiliations:** 1 Medicine, West Virginia University School of Medicine, Morgantown, USA; 2 Internal Medicine, West Virginia University School of Medicine, Morgantown, USA; 3 Internal Medicine, West Virginia University, Morgantown, USA

**Keywords:** bactetermia, candida endocarditis, endocarditis, fungemia, prosthetic aortic valves

## Abstract

Fungal endocarditis (FE) is an uncommon and life-threatening disease with unacceptably high mortality rates. Only a handful of cases of FE caused by *Candida glabrata* have been reported in the literature so far. We present a case of *C. glabrata* prosthetic valve endocarditis (PVE) in a 59-year-old woman with a history of prosthetic aortic valve who was admitted after outpatient blood cultures came positive for yeast (later identified as *C. glabrata*). A trans-esophageal echocardiogram showed vegetation on the prosthetic aortic valve. The patient was successfully treated with intravenous (IV) micafungin as she was deemed unfit for valve replacement surgery due to multiple high-risk comorbidities. The patient was treated with six weeks of IV micafungin followed by oral fluconazole for lifelong suppression, and she remained well at follow-up six months later. In this report, we review the latest literature on FE and discuss the role of antifungal therapy in its management.

## Introduction

Fungal endocarditis (FE) is a rare life-threatening condition accounting for 1-3% of all cases of infective endocarditis (IE) and 3% of prosthetic valve cases [1-4]. The management of FE typically involves a combined approach of early valve replacement surgery of the infected valves and a long course of antifungal medications [5]. In the recent literature, few studies have questioned the necessity of surgical intervention as their data showed no difference in mortality rates between the medical therapy alone group versus adjunctive surgical therapy group [6,7]. We are reporting here a patient who was a poor surgical candidate and was successfully managed with medical therapy alone (intravenous micafungin followed by oral fluconazole).

## Case presentation

We present a case of a 59-year-old woman with a past medical history of aortic stenosis status post (s/p) transcatheter aortic valve replacement (TAVR) three years ago, diastolic heart failure, end-stage renal disease (ESRD) on hemodialysis, hypertension, paroxysmal atrial fibrillation, severe pulmonary hypertension, insulin-dependent type 2 diabetes mellitus, chronic obstructive pulmonary disease (COPD) on 5 L of oxygen via nasal cannula, mild coronary artery disease, and history of colon cancer s/p partial colectomy (currently, no ostomy) who was admitted to the hospital after her blood cultures drawn at an outpatient dialysis facility were positive for yeast. She had fevers, chills, and pain at the right upper extremity dialysis arteriovenous fistula (RUE AVF) site for a few days prior to obtaining blood cultures. On physical examination, mild erythema and tenderness were noted on the RUE AVF site but no warmth or swelling appreciated. Initial laboratory results showed elevated C-reactive protein and sedimentation rate, suggestive of an active inflammatory process. The laboratory results were as follows (Table 1).

**Table 1:** Laboratory results

Test	Results	Normal range
Hemoglobin	9.6 g/dL	11.5-16.0 g/dL
Blood urea Nitrogen (BUN)	20 mg/dL	8-25 mg/dL
Creatinine	5.2 mg/dL	0.60-1.05 mg/dL
C-reactive protein (CRP)	50.2 mg/L	<8.0 mg/L
Erythrocyte sedimentation rate (ESR)	90 mm/hr	0-20 mm/hr

The patient was otherwise hemodynamically stable with stable vital signs.

**Figure 1:**
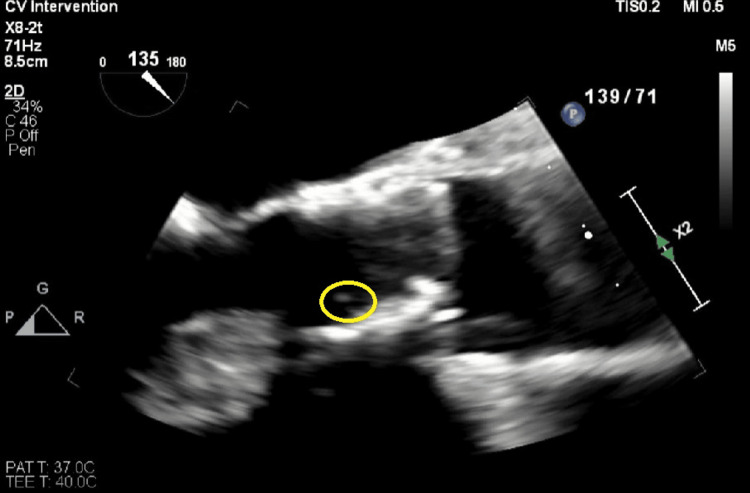
A transesophageal echocardiogram image showing an echo density measuring 1.1 x 0.2 cm (yellow circle) on the prosthetic aortic valve, consistent with a vegetation.

**Figure 2:**
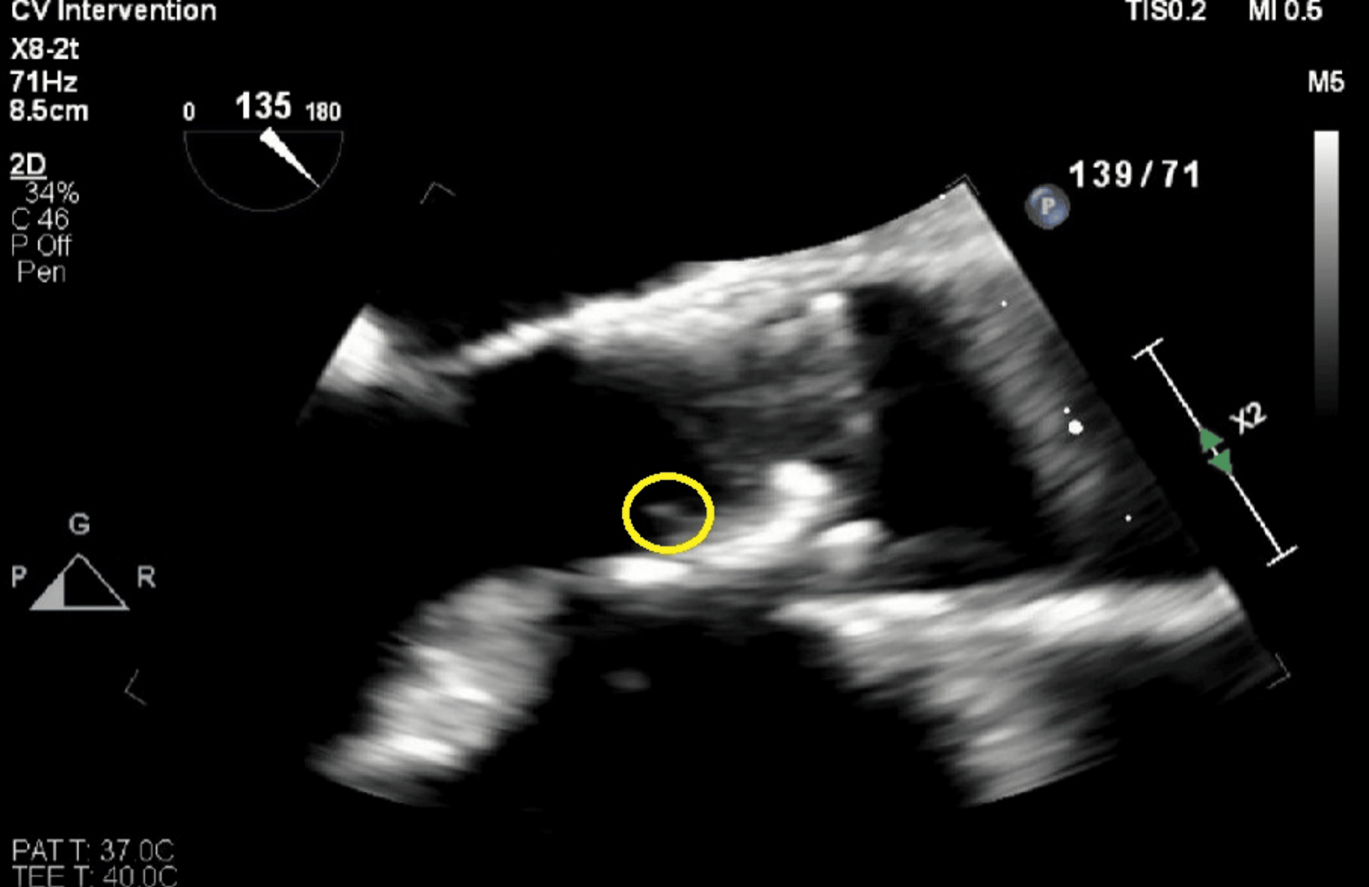
Another transesophageal echocardiogram (TEE) imaging confirming the presence and location of vegetation on the prosthetic aortic valve (yellow circle).

For her fungemia, she was started on IV micafungin 100 mg daily based on blood culture sensitivity results and in consultation with the Infectious Disease (ID). Due to concerns about a fistula infection, vascular surgery was consulted, and a fistula duplex was completed to confirm flow. It was negative for fluid collections or abscesses. Repeat blood cultures were positive for yeast twice, and the organism was identified as *Candida glabrata*. A transthoracic echocardiogram (TTE) to evaluate the TAVR valve was negative for vegetation. Due to the history of TAVR and persistently positive blood cultures for fungemia, a transesophageal echocardiogram (TEE) was performed to rule out infectious endocarditis (IE). The TEE showed a 1.1 x 0.2 cm mobile density on TAVR with leaflet restriction, concerning vegetation (Figures 1, 2, Video 1).

**Video 1:** Transesophageal echocardiogram (TEE) showing a mobile density on prosthetic aortic valve.

The ID recommended aortic valve replacement and long-term IV micafungin. The patient was evaluated by cardiothoracic surgery for valve replacement surgery, but no surgical intervention was recommended as she was deemed a poor surgical candidate due to high-risk multiple comorbidities.

Blood cultures were checked every 48 hours, and the first two blood cultures set remained positive for candidemia. The third blood culture on day 5 of hospitalization while on IV micafungin became negative. The patient remained clinically and hemodynamically stable throughout the hospitalization. The ID recommended six weeks of IV micafungin 100 mg daily followed by lifelong suppression with antifungals on discharge. Outpatient ID clinic follow-up after three and six months of discharge documented that the patient remained asymptomatic without recurrence of fever, chills, or other signs of infection. She was maintained on suppressive antifungal therapy with fluconazole (800 mg every Monday, Wednesday, and Friday after dialysis). The patient also had a three-month follow-up with cardiology, and a repeat echocardiogram was not performed as the patient was clinically asymptomatic.

## Discussion

Bacterial pathogens are the primary cause of IE, whereas fungal infections account for approximately 1-3% of cases but generally result in a more unfavorable prognosis. Fungal prosthetic valve endocarditis (PVE) is an extremely severe form of IE, with poor prognosis and high mortality rates ranging between 57% and 62.5 % as per recent studies [6,8]. *Candida *species are responsible for over 50% of fungal endocarditis cases, followed by *Aspergillus* and *Histoplasma* species. *Candida albicans* is the most frequent causative agent, and non-*albicans* species include *C. parapsilosis, C. tropicalis*, and* C. glabrata* [9]. Important risk factors include prosthetic valves, prior heart surgery, intravenous drug abuse, prolonged antibiotics therapy, parenteral nutrition, prolonged use of steroids, or other immunosuppressed states [10].

The diagnosis of FE is challenging due to its non-specific presentation, often mimicking other conditions such as bacterial endocarditis or even influenza. Symptoms can include fever (often prolonged, exceeding two weeks), chills, night sweats, malaise, and weight loss [11]. These symptoms overlap considerably with those of bacterial endocarditis, leading to delays in appropriate antifungal therapy. Embolic events are frequently observed, particularly in cases of *Aspergillus* endocarditis, and the brain, spleen, and kidneys are the common sites of embolic involvement.

Blood cultures, while a cornerstone of diagnosis, often yield positive results in fewer than 50% of cases [12], particularly with *Candida* species, due to the slow growth and fastidious nature of these organisms. Furthermore, the presence of prior antibiotic therapy can suppress fungal growth in blood cultures, leading to false-negative results

Newer laboratory tests available to detect candidemia are mannan antigen and antibody with a sensitivity of 83% and specificity of 86%, 1,3 b-D-glucan with a sensitivity of 69.9% and specificity of 87.1%, and galactomannan (helps in the diagnosis of aspergillus endocarditis) [4]. 

Another important diagnostic tool is an echocardiogram. The European Society of Cardiology guidelines recommend TTE as the first-line imaging modality, regardless of the organism. TEE is recommended in patients with prosthetic valves or cases of high suspicion despite negative TTE findings [5]. In our case, TEE was proven to be diagnostic and showed vegetation while TTE was unrevealing.

A multidisciplinary approach is required for the management of FE as it is difficult to treat due to the ability of the *Candida *species to form biofilms on native and prosthetic valves and poor penetration of antifungal agents [13]. Both the Infectious Disease Society of America and the European Society of Clinical Microbiology and Infectious Diseases guidelines recommend early valve replacement surgery of infected valves (natural or prosthetic) and a long course of antifungal medications [4,5,11]. In patients who are poor surgical candidates for valve replacement, several studies recommend initial induction antifungal therapy followed by lifelong suppression therapy [4,5,14]. All these recommendations are based on small observational studies, reports, and even expert opinions. Ellis et al. studied fungal endocarditis cases (270 patients) from 1965 to 1985 and concluded better outcomes (55% 1-year survival) in patients who received combined surgery and antifungal therapy as compared to those who had medical therapy alone (36% one-year survival) [15]. Steinbach et al. reviewed the management of *Candida* endocarditis patients published from 1996 to 2002, and 163 patients met his inclusion criteria. He concluded lower odds of death in patients who had adjunctive surgery as compared to the medical therapy alone group, although his findings did not reach statistical significance [14]. On the other hand, recently, Arnold et al.'s study (70 patients) compared both the medical therapy alone group and adjunctive surgical group, and no difference in within-hospital mortality (38% versus 34%; P = 0.77) or one-year mortality (66% versus 62%; P = 0.76) was noted [7]. The ESCAPE study, which evaluated the long-term prognosis of *Candida* PVE cases, did not show improved survival rates at six months in patients who had received adjunctive surgery [6]. Our patient was also successfully managed with micafungin and fluconazole alone without surgery and remained well at follow-up six months later. Due to this conflicting data and lack of consensus, further research is required.

## Conclusions

Fungal prosthetic valve endocarditis (PVE) is an extremely severe form of infective endocarditis with a poor prognosis and high mortality rates. The current literature recommends a combination of early valve replacement surgery of the infected valves (natural or prosthetic) and long-term antifungal treatment. Most recent studies show conflicting data without any significant mortality benefits in patients managed with antifungal therapy alone as compared to the patients who had received adjunctive surgical therapy. Further research with multicenter randomized trials or larger prospective studies is warranted to develop consensus guidelines for this deadly condition.
